# Supplementary material for: Causal evidence for a domain-specific role of left superior frontal sulcus in human perceptual decision-making
Source: eLife. 2026 Jan 30;13:RP94576. doi: 10.7554/eLife.94576 (PMC12858167; doi:10.7554/eLife.94576)
Supplement: Supplementary file 1. — All p-values are FWE-corrected for the whole brain. [file elife-94576-supp1.docx]

| Region | Peak- Side | Cluster Size | x | y | z | Z score | T score | p-value |
| --- | --- | --- | --- | --- | --- | --- | --- | --- |
| Fusiform gyrus | L | 1072 | -33 | -52 | -17 | Inf. | 22.61 | < 0.001 |
| Fusiform gyrus | R | 1523 | 33 | -52 | -11 | 7.23 | 17.24 | < 0.001 |
| Anterior intraparietal sulcus | L | 845 | -48 | -34 | 46 | 7.13 | 16.53 | < 0.001 |
| Caudal supplementary motor | L | 525 | -3 | 14 | 49 | 7.05 | 15.98 | < 0.001 |
| Middle cingulate cortex | L | 99 | -6 | -31 | 28 | 6.92 | 15.17 | < 0.001 |
| Anterior insula | L | 219 | -33 | 23 | 1 | 6.62 | 13.49 | < 0.001 |
| Hippocampus/Thalamus | R | 42 | 21 | -28 | 4 | 6.40 | 12.36 | < 0.001 |
| Hippocampus/Thalamus | L | 483 | -21 | -31 | 1 | 6.35 | 12.11 | < 0.001 |
| Anterior insula | R | 187 | 30 | 26 | -2 | 6.18 | 11.36 | < 0.001 |
| Somatosensory | L | 55 | -36 | -4 | 10 | 5.89 | 10.18 | < 0.001 |
| Cerebellum | R | 32 | 0 | -52 | -35 | 5.69 | 9.45 | < 0.001 |
| Cerebellum | L | 18 | -9 | -76 | -26 | 5.33 | 8.29 | < 0.001 |
| Premotor cortex / Inferior frontal sulcus | R | 25 | 51 | 8 | 28 | 5.32 | 8.25 | < 0.001 |
| Premotor cortex / Inferior frontal sulcus | L | 33 | -54 | 8 | 34 | 5.25 | 8.06 | < 0.001 |
| Cerebellum | R | 3 | 9 | -76 | -38 | 5.16 | 7.80 | 0.004 |
| Frontal eye field | R | 19 | 24 | -1 | 49 | 5.14 | 7.73 | < 0.001 |
| Cerebellum | L | 4 | -6 | -79 | -41 | 5.12 | 7.69 | 0.002 |
| Medial temporal lobe | L | 3 | -27 | -4 | -32 | 5.12 | 7.68 | 0.004 |
| Dorsolateral prefrontal | R | 6 | 42 | 32 | 25 | 5.09 | 7.59 | 0.001 |
| Cerebellum | R | 5 | 15 | -55 | -47 | 5.03 | 7.44 | 0.001 |
| Cerebellum | R | 3 | 18 | -67 | -47 | 4.94 | 7.18 | 0.004 |
| Cerebellum | R | 1 | 9 | -73 | -23 | 4.86 | 6.99 | 0.015 |
| Basal ganglia | L | 4 | -15 | 5 | 4 | 4.86 | 6.98 | 0.002 |
| Frontal eye field | L | 1 | -30 | -4 | 49 | 4.83 | 6.92 | 0.015 |
| Supplementary motor cortex | L | 2 | -6 | -4 | 61 | 4.83 | 6.92 | 0.007 |
| Somatosensory cortex | L | 1 | -54 | -19 | 19 | 4.82 | 6.88 | 0.015 |
| Brainstem | R | 1 | 9 | -34 | -32 | 4.80 | 6.85 | 0.015 |
| Brainstem | R | 1 | 0 | -31 | -38 | 4.80 | 6.85 | 0.015 |
| Parietal cortex | L | 1 | -24 | 2 | 55 | 4.79 | 6.81 | 0.015 |
