# Supplementary material for: Causal evidence for a domain-specific role of left superior frontal sulcus in human perceptual decision-making
Source: eLife. 2026 Jan 30;13:RP94576. doi: 10.7554/eLife.94576 (PMC12858167; doi:10.7554/eLife.94576)
Supplement: Supplementary file 2. — All p-values are FWE-corrected for the whole brain. SVC = small-volume correction. [file elife-94576-supp2.docx]

| Region | Peak- Side | Cluster Size | x | y | Z | Z score | T score | p-value |
| --- | --- | --- | --- | --- | --- | --- | --- | --- |
| Value-based choice trials > Perceptual choice trials | | | | | | | | |
| Angular gyrus | L | 344 | -45 | -70 | 43 | 5.74 | 9.62 | < 0.001 |
| Superior frontal | L | 1560 | -18 | 32 | 46 | 5.36 | 8.40 | < 0.001 |
| Temporal parietal junction | R | 204 | 51 | -61 | 37 | 4.71 | 6.62 | < 0.001 |
| Posterior cingulate cortex | L | 1368 | -3 | -55 | 22 | 4.66 | 6.51 | < 0.001 |
| Cerebellum | R | 171 | 39 | -61 | -47 | 4.62 | 6.41 | < 0.001 |
| Orbitofrontal cortex | L | 130 | -39 | 38 | -11 | 5.31 | 8.24 | < 0.001 |
| Middle temporal gyrus | L | 118 | -63 | -22 | -20 | 4.40 | 5.90 | 0.002 |
| Superior frontal | R | 75 | 18 | 35 | 46 | 3.95 | 5.02 | 0.042 |
| Medial prefrontal cortex | L | 24 | -9 | 50 | 1 | 4.02 | 5.14 | 0.027 |
| Basal forebrain | L | 11 | -15 | 20 | -14 | 3.57 | 4.33 | 0.011^SVC^ |
| Superior temporal gyrus | L | 1 | -63 | -22 | -5 | 3.19 | 3.73 | 0.033 ^SVC^ |
| Perceptual choice trials > Value-based choice trials | | | | | | | | |
| Frontal eye fields | R | 79 | 24 | -1 | 55 | 5.01 | 7.39 | 0.035 |
| Premotor cortex | R | 176 | 51 | 8 | 19 | 4.59 | 6.34 | 0.001 |
| Intraparietal sulcus | R | 646 | 39 | -40 | 46 | 4.59 | 6.34 | < 0.001 |
| Inferior temporal sulcus | R | 124 | 45 | -55 | 1 | 4.36 | 6.82 | 0.005 |
| Anterior parietal sulcus | L | 319 | -51 | -31 | 40 | 4.29 | 5.67 | < 0.001 |
