# Supplementary material for: Causal evidence for a domain-specific role of left superior frontal sulcus in human perceptual decision-making
Source: eLife. 2026 Jan 30;13:RP94576. doi: 10.7554/eLife.94576 (PMC12858167; doi:10.7554/eLife.94576)
Supplement: Supplementary file 3. — Note: Trialwise AE during both types of choices correlated negatively with BOLD activity in intraparietal sulcus (IPS) (peak at 𝑥 = −33, 𝑦 = −49, 𝑧 = 58; 𝑆𝑉𝐶 < 0.05; Figure 4—figure supplement 1C) and bilateral fusiform gyrus (right peak at 𝑥 = 33, 𝑦 = −49, 𝑧 = −14; left peak at 𝑥 = −30, 𝑦 = −52, 𝑧 = −11; FWE-corrected with cluster-forming thresholds at 𝑇(19) >2.9; Figure 4—figure supplement 1C). Note that the inverse of total evidence is directly proportional to the efficiency of evidence accumulation (see Methods for more details; SVC = small-volume correction). [file elife-94576-supp3.docx]

| Region | Peak- Side | Cluster Size | x | y | z | Z score | T score | p-value |
| --- | --- | --- | --- | --- | --- | --- | --- | --- |
| Total Accumulation Value-based Decisions | | | | | | | | |
| Lingual gyrus | L | 1338 | -9 | -88 | -2 | 5.83 | 9.98 | < 0.001 |
| Supplementary Motor Area | L | 887 | -6 | -4 | 43 | 4.58 | 6.32 | < 0.001 |
| Primary Auditory Cortex | R | 145 | 36 | -31 | 16 | 4.53 | 6.19 | < 0.001 |
| Total Accumulation Perceptual Decisions | | | | | | | | |
| Cuneus, V3 | R | 1215 | 0 | -91 | 13 | 5.00 | 7.36 | < 0.001 |
| Lingual gyrus | R | 1006 | 12 | -76 | -5 | 4.87 | 7.01 | < 0.001 |
| Superior frontal sulcus* | L | 1 | -21 | 26 | 37 | 3.13 | 3.64 | 0.039^SVC^ |
| Total Accumulation Value-based ∩ Perceptual Decisions | | | | | | | | |
| Cuneus, V3 | R | 1528 | 6 | -88 | 10 | 6.99 | 15.60 | < 0.001 |
| Postcentral gyrus | R | 316 | 6 | -49 | 73 | 4.31 | 7.73 | < 0.001 |
| Lingual gyrus* | L | 518 | -9 | -91 | 1 | 4.38 | 5.98 | < 0.001 |
| Total Accumulation Value-based > Perceptual Decisions | | | | | | | | |
| Nucleus Accumbens | R | 395 | 9 | 11 | -11 | 3.92 | 4.94 | < 0.001 |
| Supramarginal gyrus | L | 166 | -63 | -28 | 37 | 3.42 | 4.09 | 0.023 |
| Ventromedial prefrontal cortex* | L | 2 | 0 | 38 | -14 | 3.16 | 3.68 | 0.032^SVC^ |
