# Supplementary material for: Causal evidence for a domain-specific role of left superior frontal sulcus in human perceptual decision-making
Source: eLife. 2026 Jan 30;13:RP94576. doi: 10.7554/eLife.94576 (PMC12858167; doi:10.7554/eLife.94576)
Supplement: Supplementary file 4. [file elife-94576-supp4.docx]

| Region | Peak- Side | Cluster Size | x | y | z | Z score | T score | p-value |
| --- | --- | --- | --- | --- | --- | --- | --- | --- |
| Accumulation Rate for Value-based Decisions | | | | | | | | |
| Fusiform gyrus | R | 564 | 33 | -55 | -11 | 4.92 | 7.15 | < 0.001 |
| Fusiform gyrus | L | 718 | -27 | -70 | -11 | 4.92 | 7.14 | < 0.001 |
| Occipital | R | 349 | 30 | -85 | 10 | 3.75 | 4.65 | < 0.001 |
| Intraparietal sulcus | L | 58 | -28 | -66 | 38 | 4.35 | 5.80 | 0.002^SVC^ |
| Intraparietal sulcus | R | 19 | 27 | -61 | 43 | 3.52 | 4.25 | 0.032^SVC^ |
| Accumulation Rate for Perceptual Decisions | | | | | | | | |
| Fusiform gyrus | L | 1095 | -24 | -82 | -8 | 5.72 | 9.56 | < 0.001 |
| Fusiform gyrus | R | 1250 | 33 | -55 | -11 | 5.06 | 7.51 | < 0.001 |
| Intraparietal sulcus | R | 58 | 27 | -61 | 43 | 3.54 | 4.29 | 0.009^SVC^ |
| Accumulation Rate for Value-based ∩ Perceptual Decisions | | | | | | | | |
| Fusiform gyrus | L | 1151 | -24 | -82 | -8 | 5.75 | 9.69 | < 0.001 |
| Fusiform gyrus | R | 1369 | 33 | -55 | -11 | 5.47 | 8.73 | < 0.001 |
| Intraparietal sulcus | R | 81 | 27 | -61 | 43 | 3.66 | 4.49 | 0.003^SVC^ |
|  |  |  |  |  |  |  |  |  |
