# Supplementary material for: Causal evidence for a domain-specific role of left superior frontal sulcus in human perceptual decision-making
Source: eLife. 2026 Jan 30;13:RP94576. doi: 10.7554/eLife.94576 (PMC12858167; doi:10.7554/eLife.94576)
Supplement: Supplementary file 5. — Significance: *p < 0.05, **p < 0.01. [file elife-94576-supp5.docx]

|  | Perceptual | | Value-Based | |
| --- | --- | --- | --- | --- |
|  | (a) | (b) | (c) | (d) |
| Accuracy |  |  |  |  |
| (1) active DV | 0.5596*** | 0.5178*** | 0.249*** | 0.245*** |
|  | (0.053) | (0.047) | (0.061) | (0.062) |
|  |  |  |  |  |
| (2) passive DV | 0.0232 | 0.0258 | 0.006 | 0.005 |
|  | (0.178) | (0.015) | (0.026) | (0.027) |
|  |  |  |  |  |
| (3) active overall value (OV) | –0.2023*** | –0.210*** | 0.088 | 0.073 |
|  | (0.041) | (0.046) | (0.151) | (0.057) |
|  |  |  |  |  |
| (4) passive OV | –0.060 | –0.048 | –0.027 | –0.027 |
|  | (0.060) | (0.060) | (0.063) | (0.063) |
|  |  |  |  |  |
| (5) RTs |  | –1.0679*** |  | –0.254 |
|  |  | (0.160) |  | (0.165) |
|  |  |  |  |  |
| (6) Constant | 0.720*** | 2.131*** | 0.200 | 0.569 |
|  | (0.221) | (0.301) | (0.239) | (0.293) |
|  |  |  |  |  |
| RTs |  |  |  |  |
| (1) active DV | –0.058*** | –0.041*** | –0.016* | –0.013* |
|  | (0.007) | (0.007) | (0.033) | (0.006) |
|  |  |  |  |  |
|  |  |  |  |  |
| (2) passive DV | 0.002 | 0.002 | –0.003 | –0.003 |
|  | (0.002) | (0.002) | (0.004) | (0.003) |
|  |  |  |  |  |
| (3) active OV | –0.002 | –0.008 | –0.057*** | –0.056*** |
|  | (0.007) | (0.007) | (0.004) | (0.007) |
|  |  |  |  |  |
| (4) passive OV | 0.011 | 0.009 | 0.006 | 0.006 |
|  | (0.007) | (0.007) | (0.007) | (0.006) |
|  |  |  |  |  |
| (5) Choice response |  | –0.220*** |  | –0.080*** |
|  |  | (0.020) |  | (0.017) |
|  |  |  |  |  |
| (7) Constant | 1.289 | 1.442 | 1.431 | 0.010 |
|  | (0.0623) | (0.062) | (0.060) | (0.035) |
|  |  |  |  |  |
| Total Observations | 2,544 | 2,548 | 2,544 | 2,548 |
| Sessions | 8 | 8 | 8 | 8 |
| Subjects | 20 | 20 | 20 | 20 |
