# Supplementary material for: Causal evidence for a domain-specific role of left superior frontal sulcus in human perceptual decision-making
Source: eLife. 2026 Jan 30;13:RP94576. doi: 10.7554/eLife.94576 (PMC12858167; doi:10.7554/eLife.94576)
Supplement: Supplementary file 6. — Significance: *p < 0.05, **p < 0.01 (pre–post cTBS: Stimulation effect comparing the last two runs during pre-cTBS and the first two runs during post-cTBS; pre–post cTBS + training: Stimulation effect comparing all runs during pre-cTBS with the first two runs during post-cTBS; pre–post cTBS + control variables: The same as in (a) but we added control variables to test for robustness of the stimulation effect; pre–post cTBS + training + control variables: The same as in (b) but we added control variables to test for robustness of the stimulation effect). [file elife-94576-supp6.docx]

|  | (a) | (b) | (c) | (d) |
| --- | --- | --- | --- | --- |
|  | Pre-Post cTBS | + Training | + Control Variables | + Training & Control Variables |
| Accuracy |  |  |  |  |
| (1) Perceptual | –0.433* | –0.465** | –0.459* | –0.484** |
|  | (0.188) | (0.174) | (0.192) | (0.180) |
|  |  |  |  |  |
| (2) Value-based | 0.00298 | –0.0415 | 0.0042 | –0.040 |
|  | (0.124) | (0.104) | (0.123) | (0.104) |
|  |  |  |  |  |
| (3) DD Estimate | –0.293* | –0.273* | –0.319** | –0.288* |
|  | (0.114) | (0.135) | (0.115) | (0.136) |
|  |  |  |  |  |
| (4) Corrected | –0.075** | –0.094* | –0.087** | –0.103* |
|  | (0.027) | (0.045) | (0.030) | (0.047) |
|  |  |  |  |  |
| RTs |  |  |  |  |
| (1) Perceptual | –0.090* | –0.116** | –0.089* | –0.125** |
|  | (0.0331) | (0.0343) | (0.033) | (0.033) |
|  |  |  |  |  |
| (2) Value-based | –0.117** | –0.125** | –0.117** | –0.125** |
|  | (0.0298) | (0.0328) | (0.030) | (0.033) |
|  |  |  |  |  |
| (3) DD Estimate | 0.0265 | 0.00929 | 0.0273 | 0.010 |
|  | (0.0357) | (0.0353) | (0.035) | (0.035) |
|  |  |  |  |  |
| Perceptual Obs. | 1,272 | 1,907 | 1,272 | 1,907 |
| Value-based Obs. | 1,276 | 1,908 | 1,276 | 1,908 |
| Total Obs. | 2,548 | 3,815 | 2,548 | 3,815 |
| Sessions | 4 | 6 | 4 | 6 |
| Subjects | 20 | 20 | 20 | 20 |
