# Supplementary material for: Causal evidence for a domain-specific role of left superior frontal sulcus in human perceptual decision-making
Source: eLife. 2026 Jan 30;13:RP94576. doi: 10.7554/eLife.94576 (PMC12858167; doi:10.7554/eLife.94576)
Supplement: Supplementary file 7. — Significance: *p < 0.05, **p < 0.01 (pre–post cTBS: Stimulation effect comparing the last two runs during pre-cTBS and the first two runs during post-cTBS; pre–post cTBS + training: Stimulation effect comparing all runs during pre-cTBS with the first two runs during post-cTBS; pre–post cTBS + control variables: The same as in (a) but we added control variables to test for robustness of the stimulation effect; pre–post cTBS + training + control variables: The same as in (b) but we added control variables to test for robustness of the stimulation effect). [file elife-94576-supp7.docx]

|  | (a) | (b) | (c) | (d) |
| --- | --- | --- | --- | --- |
|  | Pre-Post cTBS | + Training | + Control Variables | + Training & Control Variables |
| Accuracy |  |  |  |  |
| (1) PDM SFS x Stimulation | –0.145** | –0.133** | –0.195** | –0.318** |
|  | (0.047) | (0.043) | (0.057) | (0.106) |
|  |  |  |  |  |
| (2) VDM SFS x Stimulation | 0.148 | 0.104 | 0.167 | –0.065 |
|  | (0.084) | (0.080) | (0.090) | (0.053) |
|  |  |  |  |  |
| (3) SFS x Stimulation x Task | –0.293** | –0.237** | –0.356** | –0.318** |
|  | (0.101) | (0.088) | (0.121) | (0.103) |
|  |  |  |  |  |
| (4) Corrected Triple Interaction | –0.086* | –0.093* | –0.102* | –0.131** |
|  | (0.034) | (0.038) | (0.040) | (0.047) |
|  |  |  |  |  |
| RTs |  |  |  |  |
| (1) PDM SFS x Stimulation | –0.003 | –0.005 | –0.009 | –0.005 |
|  | (0.0140) | (0.0148) | (0.0137) | (0.0142) |
|  |  |  |  |  |
| (2) VDM SFS x Stimulation | –0.003 | 0.011 | –0.003 | 0.008 |
|  | (0.0227) | (0.0182) | (0.0232) | (0.0182) |
|  |  |  |  |  |
| (3) SFS x Stimulation x Task | –0.0002 | –0.016 | –0.018 | –0.012 |
|  | (0.0273) | (0.0241) | (0.0240) | (0.0238) |
|  |  |  |  |  |
| Perceptual Obs. | 1,272 | 1,907 | 1,272 | 1,907 |
| Value-based Obs. | 1,276 | 1,908 | 1,276 | 1,908 |
| Total Obs. | 2,548 | 3,815 | 2,548 | 3,815 |
| Sessions | 4 | 6 | 4 | 6 |
| Subjects | 20 | 20 | 20 | 20 |
