# Supplementary material for: Causal evidence for a domain-specific role of left superior frontal sulcus in human perceptual decision-making
Source: eLife. 2026 Jan 30;13:RP94576. doi: 10.7554/eLife.94576 (PMC12858167; doi:10.7554/eLife.94576)
Supplement: Supplementary file 8. — Drift values by evidence level summarise the implied δc,s,i=kc,s×Ec,s,i\begin{document}$\delta _{c,s,i}=k_{c,s}\times E_{c,s,i}$\end{document} for each bin; separate drift parameters were not estimated for each evidence level. [file elife-94576-supp8.docx]

|  | $\delta$ | | $\alpha$ | | $\tau$ | | DIC |
| --- | --- | --- | --- | --- | --- | --- | --- |
| **Perceptual** | mean | SD | mean | SD | mean | SD |  |
| All | 0.388 | 0.035 | 1.848 | 0.065 | 0.522 | 0.029 | 4048.675 |
| Pre-stimulation | 0.401 | 0.041 | 1.915 | 0.078 | 0.537 | 0.035 | 2103.361 |
| Post-stimulation | 0.394 | 0.039 | 1.708 | 0.070 | 0.545 | 0.023 | 1811.714 |
|  |  |  |  |  |  |  |  |
| Evidence Level (all) | | | | | | | |
| 1 | 0.502 | 0.075 | 1.755 | 0.071 | 0.565 | 0.038 | 1443.749 |
| 2 | 0.847 | 0.108 | 1.734 | 0.062 | 0.576 | 0.029 | 1111.493 |
| 3 | 1.176 | 0.126 | 1.824 | 0.076 | 0.530 | 0.030 | 898.764 |
| 4 | 1.570 | 0.143 | 1.945 | 0.089 | 0.523 | 0.027 | 599.467 |
|  |  |  |  |  |  |  |  |
| Evidence Level (pre-stimulation) | | | | | | | |
| 1 | 0.521 | 0.083 | 1.815 | 0.088 | 0.571 | 0.043 | 785.636 |
| 2 | 0.907 | 0.122 | 1.738 | 0.080 | 0.633 | 0.044 | 576.199 |
| 3 | 1.211 | 0.159 | 1.908 | 0.096 | 0.533 | 0.036 | 494.364 |
| 4 | 1.673 | 0.183 | 2.070 | 0.116 | 0.542 | 0.036 | 324.876 |
|  |  |  |  |  |  |  |  |
| Evidence Level (post-stimulation) | | | | | | | |
| 1 | 0.516 | 0.110 | 1.634 | 0.086 | 0.596 | 0.036 | 675.095 |
| 2 | 0.865 | 0.164 | 1.651 | 0.089 | 0.573 | 0.027 | 520.592 |
| 3 | 1.207 | 0.150 | 1.720 | 0.097 | 0.547 | 0.031 | 429.265 |
| 4 | 1.512 | 0.144 | 1.764 | 0.110 | 0.537 | 0.031 | 295.764 |
