# Supplementary material for: Causal evidence for a domain-specific role of left superior frontal sulcus in human perceptual decision-making
Source: eLife. 2026 Jan 30;13:RP94576. doi: 10.7554/eLife.94576 (PMC12858167; doi:10.7554/eLife.94576)
Supplement: Supplementary file 9. — As in Supplementary file 8, evidence-level δ values are descriptive summaries of δc,s,i=kc,s×Ec,s,i\begin{document}$\delta _{c,s,i}=k_{c,s}\times E_{c,s,i}$\end{document} at each evidence level, not independently fitted drift parameters. [file elife-94576-supp9.docx]

|  | $\delta$ | | $\alpha$ | | $\tau$ | | DIC |
| --- | --- | --- | --- | --- | --- | --- | --- |
| **Value-Based** | mean | SD | mean | SD | mean | SD |  |
| All | 0.217 | 0.020 | 1.799 | 0.057 | 0.579 | 0.029 | 4960.166 |
| Pre-stimulation | 0.219 | 0.021 | 1.798 | 0.062 | 0.627 | 0.033 | 2523.472 |
| Post-stimulation | 0.222 | 0.022 | 1.707 | 0.066 | 0.585 | 0.031 | 2305.875 |
|  |  |  |  |  |  |  |  |
| Evidence Level (all) | | | | | | | |
| 1 | 0.386 | 0.054 | 1.680 | 0.064 | 0.616 | 0.033 | 1415.309 |
| 2 | 0.717 | 0.076 | 1.882 | 0.062 | 0.604 | 0.035 | 1274.095 |
| 3 | 0.806 | 0.088 | 1.748 | 0.076 | 0.618 | 0.038 | 1153.058 |
| 4 | 0.888 | 0.111 | 1.849 | 0.079 | 0.579 | 0.031 | 1135.478 |
|  |  |  |  |  |  |  |  |
| Evidence Level (pre-stimulation) | | | | | | | |
| 1 | 0.453 | 0.086 | 1.682 | 0.060 | 0.690 | 0.052 | 724.036 |
| 2 | 0.726 | 0.084 | 1.756 | 0.080 | 0.660 | 0.033 | 655.603 |
| 3 | 0.838 | 0.096 | 1.707 | 0.083 | 0.675 | 0.044 | 585.722 |
| 4 | 0.848 | 0.134 | 1.798 | 0.083 | 0.639 | 0.037 | 604.951 |
|  |  |  |  |  |  |  |  |
| Evidence Level (post-stimulation) | | | | | | | |
| 1 | 0.350 | 0.078 | 1.585 | 0.077 | 0.620 | 0.038 | 675.120 |
| 2 | 0.737 | 0.111 | 1.631 | 0.072 | 0.627 | 0.043 | 590.912 |
| 3 | 0.818 | 0.128 | 1.619 | 0.094 | 0.650 | 0.048 | 544.877 |
| 4 | 0.960 | 0.109 | 1.721 | 0.085 | 0.609 | 0.044 | 538.493 |
