# Supplementary material for: Causal evidence for a domain-specific role of left superior frontal sulcus in human perceptual decision-making
Source: eLife. 2026 Jan 30;13:RP94576. doi: 10.7554/eLife.94576 (PMC12858167; doi:10.7554/eLife.94576)
Supplement: Supplementary file 10. [file elife-94576-supp10.docx]

|  | $\delta$ | | $\alpha$ | | $\tau$ | | DIC |
| --- | --- | --- | --- | --- | --- | --- | --- |
| **Subject** | mean | SD | mean | SD | mean | SD |  |
| 1 | 0.416 | 0.050 | 1.654 | 0.086 | 0.498 | 0.014 | 155.658 |
| 2 | 0.378 | 0.042 | 2.034 | 0.104 | 0.510 | 0.020 | 246.230 |
| 3 | 0.668 | 0.057 | 1.846 | 0.120 | 0.487 | 0.017 | 75.702 |
| 4 | 0.257 | 0.034 | 2.336 | 0.112 | 0.742 | 0.026 | 355.873 |
| 5 | 0.147 | 0.034 | 2.026 | 0.086 | 0.587 | 0.018 | 346.635 |
| 6 | 0.358 | 0.044 | 1.796 | 0.086 | 0.567 | 0.015 | 207.893 |
| 7 | 0.504 | 0.050 | 1.932 | 0.109 | 0.565 | 0.018 | 164.511 |
| 8 | 0.338 | 0.047 | 1.678 | 0.075 | 0.357 | 0.012 | 194.419 |
| 9 | 0.571 | 0.052 | 2.026 | 0.120 | 0.446 | 0.018 | 152.084 |
| 10 | 0.330 | 0.038 | 2.296 | 0.116 | 0.222 | 0.017 | 283.846 |
| 11 | 0.252 | 0.042 | 1.649 | 0.075 | 0.547 | 0.017 | 242.651 |
| 12 | 0.482 | 0.045 | 2.248 | 0.135 | 0.555 | 0.021 | 201.924 |
| 13 | 0.361 | 0.049 | 1.532 | 0.071 | 0.484 | 0.011 | 151.426 |
| 14 | 0.578 | 0.055 | 1.823 | 0.106 | 0.494 | 0.016 | 121.826 |
| 15 | 0.271 | 0.041 | 1.835 | 0.083 | 0.656 | 0.017 | 263.821 |
| 16 | 0.189 | 0.035 | 1.969 | 0.086 | 0.551 | 0.021 | 333.834 |
| 17 | 0.676 | 0.065 | 1.597 | 0.088 | 0.345 | 0.010 | 36.774 |
| 18 | 0.359 | 0.048 | 1.516 | 0.072 | 0.592 | 0.012 | 146.552 |
| 19 | 0.360 | 0.041 | 2.016 | 0.101 | 0.728 | 0.017 | 247.041 |
| 20 | 0.375 | 0.055 | 1.406 | 0.067 | 0.490 | 0.012 | 129.653 |
