# Supplementary material for: Causal evidence for a domain-specific role of left superior frontal sulcus in human perceptual decision-making
Source: eLife. 2026 Jan 30;13:RP94576. doi: 10.7554/eLife.94576 (PMC12858167; doi:10.7554/eLife.94576)
Supplement: Supplementary file 11. [file elife-94576-supp11.docx]

|  | $\delta$ | | $\alpha$ | | $\tau$ | | DIC |
| --- | --- | --- | --- | --- | --- | --- | --- |
| **Subject** | mean | SD | mean | SD | mean | SD |  |
| 1 | 0.165 | 0.037 | 1.582 | 0.067 | 0.612 | 0.012 | 224.450 |
| 2 | 0.280 | 0.034 | 2.076 | 0.103 | 0.606 | 0.017 | 256.215 |
| 3 | 0.085 | 0.035 | 1.610 | 0.068 | 0.526 | 0.015 | 271.958 |
| 4 | 0.097 | 0.028 | 2.158 | 0.091 | 0.684 | 0.023 | 394.165 |
| 5 | 0.257 | 0.034 | 1.961 | 0.096 | 0.721 | 0.020 | 270.684 |
| 6 | 0.198 | 0.035 | 1.646 | 0.074 | 0.518 | 0.015 | 239.416 |
| 7 | 0.187 | 0.036 | 1.680 | 0.074 | 0.642 | 0.013 | 242.186 |
| 8 | 0.090 | 0.038 | 1.559 | 0.064 | 0.330 | 0.011 | 235.979 |
| 9 | 0.220 | 0.033 | 1.950 | 0.093 | 0.560 | 0.021 | 298.440 |
| 10 | 0.184 | 0.036 | 1.667 | 0.075 | 0.584 | 0.016 | 254.361 |
| 11 | 0.137 | 0.032 | 1.794 | 0.078 | 0.479 | 0.016 | 297.167 |
| 12 | 0.226 | 0.030 | 2.236 | 0.109 | 0.735 | 0.021 | 317.836 |
| 13 | 0.185 | 0.042 | 1.389 | 0.060 | 0.483 | 0.011 | 173.978 |
| 14 | 0.291 | 0.036 | 1.963 | 0.094 | 0.580 | 0.016 | 243.953 |
| 15 | 0.342 | 0.037 | 1.997 | 0.106 | 0.663 | 0.018 | 206.759 |
| 16 | 0.160 | 0.032 | 1.964 | 0.084 | 0.643 | 0.017 | 315.890 |
| 17 | 0.288 | 0.048 | 1.431 | 0.062 | 0.338 | 0.008 | 125.692 |
| 18 | 0.379 | 0.041 | 1.805 | 0.097 | 0.592 | 0.016 | 155.570 |
| 19 | 0.273 | 0.035 | 1.853 | 0.088 | 0.759 | 0.015 | 235.053 |
| 20 | 0.331 | 0.038 | 1.871 | 0.093 | 0.516 | 0.015 | 201.545 |
